# Post-Exercise Cognition and Prefrontal Hemodynamic Responses in Athletes: An Investigation of Low vs. High Glycemic Index Breakfast

**DOI:** 10.3390/nu17203296

**Published:** 2025-10-20

**Authors:** Çiğdem Bediz, Ferya Bertan, Erkan Günay, Egemen Mancı, Cem Şeref Bediz

**Affiliations:** 1 Faculty of Health Sciences, University of Kyrenia, Kyrenia 99320, Türkiye; cigdem.bediz@kyrenia.edu.tr; 2 Department of Physiology, Dokuz Eylül University, Izmir 35220, Türkiye; 3 Department of Coaching Education, Faculty of Sport Science, Celal Bayar University, Manisa 45140, Türkiye; swimerkan@gmail.com; 4 Department of Exercise and Sport Sciences, Faculty of Health Science, İzmir Demokrasi University, Izmir 35140, Türkiye; egemenmanci@gmail.com; 5 Faculty of Medicine, University of Kyrenia, Kyrenia 99320, Türkiye

**Keywords:** glycemic index, breakfast, cognition, prefrontal hemodynamics, nutritional interventions

## Abstract

**Background/Objectives**: This study aimed to investigate the effects of low and high glycemic index (LGI and HGI) breakfasts on post-exercise cognitive functions and prefrontal hemodynamic responses. **Methods**: Ten male athletes aged 18–22 years participated in this study. The athletes conducted two laboratory visits in a randomized manner. Athletes were given different glycemic index (GI) levels (low and high) for pre-exercise meals on different days, with the same calorie values, carbohydrate, and fat content. A total of 90 min after breakfast, a 30 min submaximal exercise was performed using a cycling ergometer. During the laboratory visits, blood glucose measurements were performed at the 0th (fasting), 90th (pre-exercise), and 120th (post-exercise) min. Additionally, the “3-Back test” was performed pre- and post-exercise to assess working memory and their prefrontal hemodynamic responses were monitored via functional Near-Infrared Spectroscopy. The collected data were evaluated in the SPSS 22 statistical program. **Results**: The HGI breakfast led to higher blood glucose levels at the 90th (pre-exercise) and 120th min (post-exercise) than LGI breakfast (*p* < 0.05). No difference was observed between HGI and LGI breakfasts in the results of the “3-Back Test” performed pre- and post-exercise. In terms of prefrontal hemodynamic responses, no difference was observed in post-exercise oxy-hemoglobin responses between the conditions. **Conclusions**: The findings of the study indicate that an increase in the glycemic index of breakfast has the potential to affect prefrontal oxygenation responses during cognitive tasks. However, no effect of glycemic index level was observed on cognitive and hemodynamic values at the end of the exercise.

## 1. Introduction

Athletes’ dietary habits significantly influence their performance and health. In sports nutrition, the aim is to ensure an adequate and balanced intake of nutrients by adjusting based on the athlete’s gender, age, daily physical activity, and the specific sport they engage in, considering both training and competition periods. The contribution of nutrients to daily energy varies depending on the type of sport activity an athlete engages in. The most crucial factor influencing these proportions is whether the required energy is supplied through aerobic and/or anaerobic pathways [1,2,3]. Carbohydrates constitute the most critical macronutrient that affects performance in various types of exercise and sports, serving as the primary energy source for anaerobic metabolism and the only fuel usable for high-intensity exercises [4]. The type of carbohydrate is also essential for physical performance. Several studies have demonstrated that consuming low-glycemic-index (GI) foods pre-exercise results in more stable blood glucose levels and significantly improves performance [5,6]. Low GI increases FFA utilization during exercise. The glucose levels were higher during and after exercise. In this case, the accessibility of glucose in the brain increases after exercise. It is essential to maintain cognitive skills and physical performance.

Additionally, the brain is susceptible to nutritional changes, and there is evidence suggesting a relationship between blood glucose levels and memory improvement [7]. One of the nutritional characteristics that plays a critical role in the fluctuation in blood glucose levels is the glycemic index of ingested foods. Studies on healthy adolescents have shown that foods with different glycemic indices affect cognitive functions differently. Some studies concluded that low-glycemic-index foods have more positive effects on cognitive performance. In contrast, others have revealed that foods with high glycemic index provide short-term cognitive benefits [7,8,9]. In addition to physical strength, developing sport-specific cognitive skills such as perception, attention, decision-making, and reaction time is crucial for superior performance and tactical development [10,11]. Therefore, alongside physical training, developing cognitive skills is essential to sustain exercise and perform the cognitive activities required in sports and competitions at the highest level. Consequently, there has been an increasing trend of conducting studies to evaluate cognitive functions in athletes and identify factors that influence them. The novelty of this study lies in being among the few investigations to examine the combined effects of breakfast glycemic index and acute exercise on both cognitive performance and prefrontal hemodynamic responses. While the effects of nutrition and exercise on cognition have been examined separately, studies that evaluate their interactive influence remain scarce.

Therefore, this study aimed to investigate whether the glycemic index (GI) of a pre-exercise breakfast influences post-exercise cognitive functions and prefrontal hemodynamic responses in athletes. We hypothesized that breakfast GI would differentially modulate post-exercise cognitive performance and cerebral oxygenation, such that low-GI meals—by providing more stable glucose availability—would support sustained cognitive processing and efficient neurovascular coupling, whereas high-GI meals—through rapid glucose fluctuations—might lead to transient metabolic advantages but less stable neural activation patterns during cognitive tasks.

## 2. Materials and Methods

### 2.1. Participants

In this study, sixteen male athletes between the ages of 18 and 22 were recruited (age: 19 ± 1.5 years). Participants whose calculated VO_2_max values were above 40 mL/kg/min and body mass index (BMI) values were below 30 kg/m^2^ were considered eligible for inclusion. We excluded participants who had any neurological, psychiatric, cardiovascular, orthopedic, or endocrinological disorders and whose BMI and VO_2_max values did not meet the criteria. Four participants were excluded from the study: two had a VO_2_max below 40 mL/kg/min, another was unable to consume some of the breakfast items provided, and one person experienced a physical injury. Furthermore, two other participants expressed their desire to not continue with the study and voluntarily withdrew from it. Consequently, the study was completed with a total of 10 male athletes. The other exclusion criteria were neurologic, medical, or cardiovascular diseases, as well as the use of medication. The sample size was calculated using the G-Power 3.1.9.4 program for post hoc power analysis. We entered the means of the low glycemic index (LGI) and high glycemic index (HGI) values into the group means separately. Then, with the 10 participants (with an alpha/mistake rate of 5%), we achieved 96% power. All measurements were held between 10:30 and 12:00 a.m., and participants were not allowed to eat or drink during the experiment (e.g., caffeine, alcohol, vitamin complexes) that could affect their performance, 2–3 h before the exercise. In addition, participants were required to fast for at least 12 h before consuming the test meals. All participants were informed about the procedures, and each provided written consent. This study was approved by the Local Ethics Committee of the Dokuz Eylül University (protocol code: 403-SBKAEK, date of approval: 17 February 2017). This study conformed to the Declaration of Helsinki standards.

### 2.2. Experimental Design

**Figure 1:**
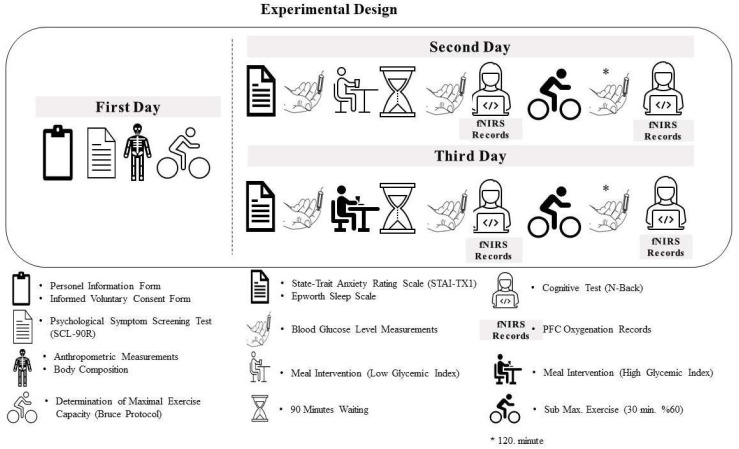
Experimental design.

The participants visited the laboratory on three different days (see Figure 1) and were randomly assigned according to their order of registration for the study. On the first day, participants were informed about the study, familiarized with the environment, and written consent was obtained. In addition, on the day of the laboratory, before the measurement, “Symptom Checklist (SCL-90R)” was used to assess the participants’ general mental state, “State-Trait Anxiety Inventory Form (STAI-TX1)” was used to determine their anxiety, and the “Epworth Sleepiness Scale (ESS)” was used to ensure that there were no other factors affecting the results of the study. On the same day, body composition measurements (body weight, fat mass, muscle mass, and fat-free mass) were performed using a laboratory-type InBody 720 (Body Composition Analyzers, Seoul, Republic of Korea) [12] bioelectrical impedance device. Then, the Bruce protocol was applied to determine the maximal oxygen consumption of the participants [13,14,15,16]. On the second and third days, the participants came to the laboratory under fasting conditions. After their blood glucose levels were checked (0th min), they were given low glycemic or high glycemic index breakfasts. Blood glucose levels were measured 90 min after breakfast (pre-exercise).

The Psychology Experiment Building Language (PEBL) test battery was used to administer the cognitive test (3-Back Test) [17,18,19]. Functional Near-Infrared Spectroscopy (fNIRS) was used to assess the oxy-hemoglobin concentrations in the prefrontal cortex (PFC) during the cognitive test. After the cognitive test was completed, the participants performed 30 min of submaximal cycling exercise. After this exercise protocol, blood glucose levels were measured again, and the cognitive test was repeated together with the fNIRS measurement (post-exercise). On the third day, the same procedures were repeated, with only the glycemic index value at breakfast changed. The whole experimental setup is presented in Figure 1.

### 2.3. Measurement of Blood Glucose Levels

Blood glucose measurements were performed at the 0th (fasting), 90th (pre-exercise), and 120th min (post-exercise). Blood samples were collected from the fingertip and analyzed using a portable glucometer (FreeStyle Optium™ Neo H Glucometers; Abbott Healthcare, Avon, MA, USA).

### 2.4. Nutritional Intervention Planning: High Glycemic Index (HGI) and Low-Glycemic-Index (LGI) Breakfast Menus

Participants were asked to fast for at least 12 h before consuming a meal with a different glycemic index. Breakfast was preferred as the meal in the study because the glycemic response is more pronounced in the morning hours. The energy value of breakfast was determined as 25% of the daily energy requirement. The basal metabolic rate (BMR) of the participants was calculated separately using both the Harris–Benedict method [20], which uses height and weight parameters, and the Cunningham method [21,22], which is based on lean body mass, and an average value was found. Daily energy requirements were determined by adding the physical activity factor to the basal metabolic rate value, based on the participants’ activity status in the “Personal Information Form”. Breakfasts with different glycemic indexes were prepared with breakfast ingredients consisting of natural foods (whole grain bread, white bread, apples, raisins, milk, feta cheese, and tomatoes). Participants were asked to consume the given meal within 15 min. After calculating the energy value of each participant’s breakfast meal, the same energy value was calculated for two products with carbohydrate, fat, and protein content but with different GI values, and different menus were planned. Energy and macronutrients were kept constant, and for similar foods (white bread versus whole grain bread, etc.), only the GI values of the foods were taken into consideration. The menus were prepared using natural foods frequently consumed in our country such as bread, tomatoes, cheese, milk, and fruits, and were presented to the participants on the 1st and 2nd test days. The energy and nutrient contents of the meals were calculated using the label information of the foods used and the “National Food Composition Database” [23], and GI values were determined according to the International Glycemic Index and Glycemic Load Tables [24,25]. Whole grain bread and apples (GI value < 55) were used in the LGI breakfast menu, white bread and raisins (GI value > 70) were used in the HGI breakfast menu, and tomatoes, cheese, and milk were used in both breakfast menus. A sample menu prepared for a participant is presented in Table 1. The meal composition of all participants was the same, as in the example in Table 1. Only the amounts of nutrients varied for each person, depending on their calorie needs.

### 2.5. Measurement of Cognitive Performance

**Figure 2:**
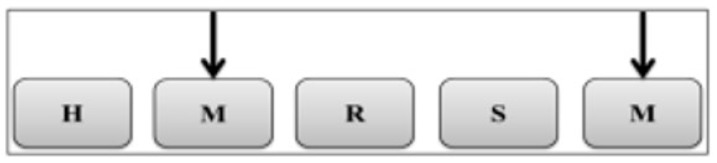
A schematic illustration of the “3-Back test.

The 3-Back test was used to measure cognitive functions (Figure 2), with the help of a laptop computer using the PEBL test battery [18,26,27]. During the test, 120 letter sequences consisting of letters H, M, R, S, M, M, S, S, R, and B were applied. The letters appeared on the computer screen for 500 ms, and the inter-stimulus interval was 1500–2000 ms. In the test, participants were asked to press the designated key on the keyboard if the letter on the screen was the same as the previous three letters. The test lasted for approximately four minutes. During the “3-Back test”, the “number of correct answers (%)” and “number of wrong answers (%)” as well as the “correct answer reaction time (ms)” and “total reaction time (ms)” data were automatically recorded on the computer. All participants completed familiarization sessions to learn about the “3-Back test” during the 90 Min post-breakfast waiting period. Then, at 90 min (pre-exercise) and 120 min (post-exercise) after breakfast, the “3-Back test” was fully administered.

The task was completed in a quiet, well-lit room with a desk, PC screen, mouse, and keyboard in their visual field. All measurements were performed on the same PC, and cognitive tests were performed on the same display. During the cognitive assessments, participants were instructed to sit 70 cm away from a 17-inch computer screen [28,29,30].

### 2.6. Exercise Protocol

The maximal exercise capacity of the participants was measured with the “Modified Bruce Protocol” using a cycling ergometer (Monark 839E, Vansbro, Sweden). The test started with a 50-watt load and 70 rpm pedal speed, and the load gradually increased every 3 min and continued until the participant was exhausted [14]. During the whole test, the heart rates of the participants were continuously monitored using a chest strap (Polar RS800, Port Washington, NY, USA). The VO_2_max levels were calculated based on the levels reached at the end of the test. On the 2nd and 3rd test days, the participants performed a submaximal exercise by pedaling at a constant rate of 30 min at a workload corresponding to 60% of their VO_2_max levels. This exercise method was selected to represent moderate-intensity aerobic activity, which is known to enhance cerebral perfusion and cognitive arousal without inducing metabolic fatigue or hypoglycemia. This intensity aligns with the established literature demonstrating optimal conditions for observing exercise-induced cognitive facilitation.

### 2.7. The Functional Near-Infrared Spectroscopy (fNIRS) Recordings

In the present study, a non-invasive and easily adaptable fNIRS device (fNIRS Devices 1100 LLC, Potomac, MD, USA) was used to monitor hemodynamic changes in the prefrontal cortex (PFC). Hemodynamic changes were recorded from 16 different channels using a sensor pad with four light sources and 10 light detectors. This sensor pad, identified as the PFC in the device’s user handbook, was placed on the participants’ foreheads as previously mentioned [29,31]. A black band was wrapped around the pad to limit ambient light. Sixteen channels recorded hemodynamic responses at a frequency of 2 Hz and a source–detector distance of 2.5 cm. The amount of light from the light source was transmitted to and sensed by the fNIRS device, and the concentration changes in oxy-hemoglobin (HbO_2_) and deoxy-hemoglobin (deoxy-Hb) in that region were estimated based on the amount of light absorbed in the cerebral tissues. The sum of these two parameters gives the total hemoglobin (total-Hb). Data processing and computations were performed using the Cognitive Optical Brain Imager (COBI) software (COBI Studio v1.5.0.40). For the offline analysis, the raw fNIRS signals were filtered in fNIRS Soft with a finite impulse response (FIR) band-pass filter set between 0.01 and 0.50 Hz to remove respiratory activity, heart rate-related fluctuations, and unrelated high-frequency noise [29,30,31]. In addition, the common average reference (CAR) technique, as described by Pfurtscheller et al. (2010) and von Lühmann et al. (2020), was applied, and the entire dataset was recalculated to further minimize the influence of physiological noise on the fNIRS measurements [32,33,34,35]. Prefrontal cortex hemodynamics were recorded simultaneously with fNIRS during the “3-Back test” performed at the 90th min (pre-exercise) and 120th min (post-exercise) after breakfast. Calculations were performed using the mean values of the 16 channels from the data obtained. Since HbO_2_ values at the beginning of the cognitive test (baseline) showed individual differences, HbO_2_ measurements were not evaluated as absolute values. Therefore, the delta difference (HbO_2_-delta_difference) during the test was calculated by taking the difference between the average HbO_2_ value in the last min of the “3-Back test” and the average value in the first min (delta calculation). In this way, it was statistically investigated whether HbO_2_ elevation during the test differed between the HGI and LGI conditions when the same cognitive test was applied. “HbO_2_-delta_difference” results were used in statistical evaluations.

### 2.8. Statistical Analysis

SPSS 22 (IBM SPSS Statistics for Windows, Version 22.0, IBM Corp., Armonk, NY, USA) was used for statistical analyses. Normality tests (Shapiro–Wilk) were used to assess the statistical distribution of the data. According to the test results, the entire dataset did not have a normal distribution. Therefore, the Wilcoxon test with Bonferroni correction was used for pairwise comparisons. In addition, a Spearman correlation test was performed to examine the relationship between blood glucose levels, hemodynamic changes, and cognitive performance. The significance level was set at *p* < 0.05.

## 3. Results

### 3.1. Demographic Information, Body Composition, and Physiologic Parameters

The variables for participants’ age, body weight, height, body mass index (BMI), fat mass, muscle mass, fat-free mass, lean tissue mass, VO_2_max, resting heart rate, and target heart rate are presented in Table 2.

### 3.2. Blood Glucose Level Results

The participants’ fasting (0th min), pre-exercise (90th min), and post-exercise (120th min) blood glucose level measurements on the test days when participants were given breakfast with HGI and LGI, along with the comparison according to the GI value of breakfast, are presented in Table 3.

### 3.3. Results of “3-Back Test”

The percentages of correct and wrong answers, along with the correct answer and total reaction time of the “3-Back test,” were compared based on the GI value and are presented in Table 4. Accordingly, high or low GI values did not create a statistically significant difference in any of the outputs of the “3-Back test.” The comparison of “3-Back test” results pre- and post-exercise is presented in Table 4. In both glycemic index values, no significant difference was found in the “3-Back test” results pre- and post-exercise.

### 3.4. HbO_2_ Measurements During Cognitive Tests

**Figure 3:**
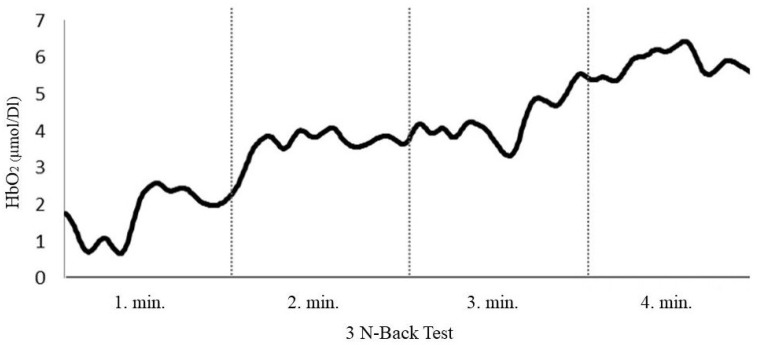
An example of the average of HbO_2_ values from 16 channels.

**Figure 4:**
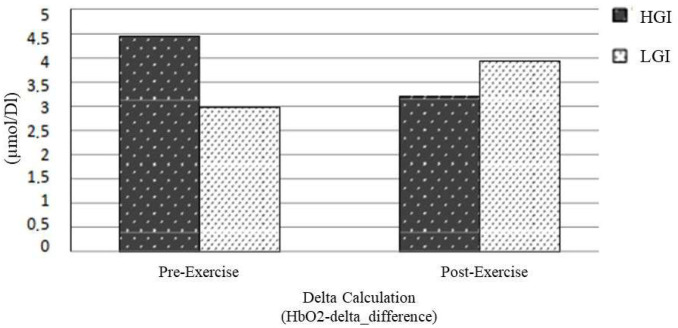
Comparison of HbO_2_-delta_difference values of participants.

The mean HbO_2_ level change during the “3-Back test” is shown in Figure 3. The HbO_2_ levels started to increase when the cognitive task started. The comparison of HbO_2_-delta_difference according to the GI value of breakfast is shown in Table 5 and Figure 4. A comparison of the HbO_2__delta_difference of the participants pre- and post-exercise is shown in Table 5.

As shown in Figure 4, although not statistically significant, HbO_2_-delta_difference decreased post-exercise for breakfast with HGI and LGI.

### 3.5. The Relationship Between Blood Glucose Levels, Changes in Brain Hemodynamics, and Results of the “3-Back Test”

The correlation between blood glucose levels after breakfast with different GI given to the participants and the change in brain hemodynamics (HbO_2_-delta_difference) during the cognitive task was also examined. Accordingly, there was a statistically significant positive correlation between the percentage of wrong answers before exercise and HbO_2_-delta_difference in LGI breakfast (r = 0.68, *p* < 0.05). In the pre- and post-exercise periods, there was no statistically significant association between blood glucose levels and brain hemodynamics and cognitive task scores.

## 4. Discussion

In this study, we examined whether the glycemic index value of breakfast consumed by athletes pre-exercise influences cognitive functions post-exercise and the hemodynamics of the brain during the cognitive task. According to the literature, our study is one of the pioneering studies examining how the combined effect of the GI value of a meal and acute exercise affects the cognitive functions and PFC hemodynamics of athletes.

### 4.1. The GI Value of Breakfast and the Combined Effect of Exercise on Blood Glucose Levels

According to the measured blood glucose results, breakfast with HGI caused a significant increase in blood glucose levels both pre- and post-exercise compared to breakfast with LGI (*p* < 0.05; Table 3). In a study by Wu and Williams [36] on the effects of breakfast with HGI and LGI on exercise performance in athletes, it was reported that a meal with LGI significantly increased blood glucose levels at the 30th, 60th, and 90th min [36]. In addition, when the LGI breakfast was given, the blood glucose level at the 90th minute was measured to have decreased below the fasting value. In the present study, the blood glucose level at the 90th minute was measured (78.00 mg/dL). The fasting value (0th min—96.40 mg/dL) was lower when the LGI breakfast was given, but this difference was not statistically significant. This was thought to be related to the fact that LGI foods later increase blood glucose levels. On the other hand, the iAUC analysis results presented in Table 6 above show that the HGI breakfast creates significantly higher overall glycemic exposure than the LGI breakfast. This indicates a different glucose breakdown pattern before exercise and is consistent with the proposed neuronal glucose availability and cerebrovascular coupling mechanism.

It is known that athletes generally tend to consume high levels of carbohydrates, and regular high-intensity exercise increases catecholamine (adrenaline and norepinephrine) concentrations, which may cause hyperglycemia post-exercise [3]. Aydın et al. (2000) found that blood glucose levels were higher after 30 min of moderate-intensity (70% VO_2_max) aerobic exercise than pre-exercise [37]. In this study, 30 min moderate-intensity exercise was performed. At the beginning of exercise, glycemia is expected to increase to meet the increased energy demand. Since the blood glucose level is a very sensitive metabolic process, regulatory mechanisms are activated rapidly and with pinpoint accuracy when it changes. Therefore, significant differences can be observed when extreme conditions are reached. Additionally, the results showed that the LGI breakfast exhibited a more stable pattern in terms of glucose availability during submaximal exercise. The fact that the LGI and HGI breakfasts showed different glucose availability patterns for the 30 min submaximal exercise volume used in the study suggests that nutritional planning based on glycemic index may be necessary for breakfast before exertion for longer submaximal tasks.

### 4.2. The GI Value of Breakfast and the Combined Effect of Exercise on Working Memory

As presented in Table 5, the results of the “3-Back test” performed pre-exercise were similar in both the HGI and LGI groups. In the present study, the differences in both GI values post-exercise, although not statistically significant, were more noticeable after breakfast. Some physiological mechanisms explaining the direct or indirect effects of the glycemic index on cognitive functions have been discussed in previous studies [38,39]. In a pioneering study on the subject, Fischer et al. focused on energy supply to nerve cells, neurotransmitter-hormone modulations, and nervous system activation to explain the effects of glycemic index values on cognition [40]. Considering this information and the increased arousal level induced by submaximal exercise, time-dependent differences in glucose availability under HGI and LGI conditions may have compensated for the metabolic and oxidative demands of prefrontal neuronal activity during cognitive tasks at similar levels through different compensation mechanisms (higher stress or efficiency in responding to demands).

Studies in the literature show that the state of arousal created by acute submaximal exercise affects cognitive functions [41]. Furthermore, Hillman et al. (2009) showed that children’s cognitive control of attention improved after 20 min of moderate-intensity walking [42]. On the other hand, Tsai et al. (2014) concluded that there is a positive interaction between moderate-intensity aerobic exercise and cognitive functions in young adults with different conditions [43]. A meta-analysis of 79 studies examining the effect of acute exercise on cognitive performance reported that light and moderate-intensity exercise positively affected cognitive performance. In contrast, high-intensity exercise had an adverse effect [44]. Looking at the literature related to glycemic index and cognition, one study indicated that a low GI may be beneficial for cognitive functions in adults [7]. Furthermore, the results of a meta-analysis study revealed that a breakfast meal composed of LGI content may benefit episodic memory and attention in the period after 120 min [45].

These results are consistent with the literature and show that submaximal exercise has a positive effect on working memory. Furthermore, the study is considered to make a unique contribution by focusing on the effect of glycemic level + exercise on cognition. The positive correlation between wrong responses and HBO_2_, as revealed by the correlation analysis results, may suggest the need for future research to identify the possible effect of the glycemic index on the exercise–cognition relationship. In addition, previous research has demonstrated that exercise not only benefits cognitive functions but also reduces stress in young adults. In line with this, Guerriero et al. (2025) reported in their scoping review that various forms of physical activity consistently decrease stress and enhance academic performance in university students. Moreover, their findings emphasize the lack of standardized intervention protocols in the current literature, which aligns with our study’s call for more clearly defined and reproducible exercise–nutrition paradigms in future research [46].

### 4.3. The GI Value of Breakfast and the Combined Effect of Exercise on Brain Hemodynamics

There is an increase in the hemodynamics of the brain during cognitive tests in both athletes and normal individuals [47,48]. In this study, an increase in HbO_2_ level was observed in all measurements during the “3-Back test”. However, calculating oxygenation values based on the delta calculation may have caused the differences in absolute values to decrease, potentially losing significance. In the correlation analysis, when the results of the “3-Back test” and HbO_2_-delta_difference data were analyzed, it was concluded that there was a significant positive correlation between the percentage of wrong responses pre-exercise and PFC oxygenation on the breakfast day with LGI (*p* < 0.05). Although the change in the HbO_2_ level of the brain is a sensitive and valid physiological measurement, there is no definite information about its connections with brain functions and changes in brain oxygenation. Despite the rapid increase in research in this field, it is difficult to compare results due to the lack of standards and normal values as well as the continuous development of techniques. In principle, it is expected that with an increase in neuronal activity or cognitive work, metabolism and oxygenation will increase. According to the results of this study, the correlation between the increase in brain oxygenation and the percentage of wrong answers may indicate that a metabolic response occurs in the brain during problem solving. According to the results of this study, the correlation between increased brain oxygenation and the percentage of wrong answers may indicate compensatory efforts under a high task load. However, this situation has not been fully clarified and needs to be examined further in future studies.

In general, an increase in cerebral blood flow, including the effect of exercise, is associated with neural oxygen utilization. However, glucose utilization is as essential as oxygen utilization in muscle tissue during exercise, and the mechanisms for supplying oxygen, compared to glucose, are known to be superior in the body. In addition, glucose is an essential metabolite of neural activity, and its utilization increases during cognitive tasks [49]. The increased glucose requirement of neurons may cause an increase in cerebral blood supply. In these two studies [34,35], which were conducted in the same laboratory as our study using the fNIRS imaging method, no nutritional intervention was performed pre-exercise, and the nutritional status and blood glucose levels were not specified. Compared to these two studies, in which higher HbO_2_ levels were found post-exercise than pre-exercise in all study groups, it was noteworthy that the increase in blood flow was lower (*p* > 0.05) on breakfast day with HGI compared to pre-exercise in our study (Figure 4).

Breakfast with HGI may have been more effective in meeting the glucose needs of the neurons, possibly due to the reduced need for blood flow. However, from the same point of view, when we examined the HbO_2_ level during the cognitive task in the pre-exercise period (Figure 4), the fact that the HbO_2_ level tended to increase more on the breakfast day with HGI suggested that it was not compatible with the post-exercise results. It was concluded that the GI value may affect the hemodynamics of the brain during the cognitive task, but in which postprandial period, in which direction, and by which mechanism, and the role of exercise in this effect in the intake of different GI meals are not clear in light of current knowledge; it is be essential to investigate these aspects in future studies. It was thought that the role of GI in brain hemodynamics could be evaluated more clearly without the effect of exercise. Since the effects of exercise on cognitive functions [49] and GI on glycemia [7] vary among individuals, our study was conducted on the same participants, unlike many other studies on this subject, to avoid the influence of personal differences.

### 4.4. Limitation

This study has several limitations. First, the planned number of participants could not be reached, resulting in a relatively small sample size (n = 10). This restricts the generalizability of the findings, and future studies with larger cohorts are expected to provide more robust results. Moreover, insulin and other metabolic hormones were not measured, which limits direct inferences regarding peripheral–central fuel dynamics. The fNIRS system used did not include short-separation channels, preventing the regression of superficial blood flow effects. Cognitive assessment was restricted to the 3-Back task, which constrained generalization of the findings to other executive domains. Additionally, employing higher-intensity exercise protocols in future studies may help to evaluate more pronounced effects on blood glucose levels. Examining the combined impact of GI value and exercise on different cognitive abilities may also contribute to clarifying the literature. Furthermore, the limited availability of raw data means that advanced statistical analysis cannot be performed, which is another limitation. Taken together, these limitations underscore the importance of conducting future research with larger samples, additional physiological measures, further analysis, and more comprehensive cognitive batteries.

## 5. Conclusions

In conclusion, our findings highlight the potential impact of breakfast glycemic index levels on brain oxygen consumption during cognitive tasks. It is believed that further research with larger participant groups is required to understand this potential better. In contrast, our study observed that the glycemic index level did not influence cognitive and hemodynamic responses after moderate-intensity exercise. Still, positive changes related to exercise were observed in the participants. These results indicate that exercise itself may serve as a robust modulator of cognitive performance, independent of pre-exercise dietary glycemic load.

The present study contributes to sports nutrition and brain hemodynamics research by being among the few examining the combined influence of breakfast GI and acute exercise on cognition. For athletes and active individuals, these findings suggest that while pre-exercise low-GI meals may help maintain stable glucose availability, engaging in moderate-intensity exercise can enhance working memory performance regardless of GI condition. Future studies with larger sample sizes, including metabolic hormone measures, diverse exercise intensities, and broader cognitive assessments, are warranted to clarify underlying mechanisms and strengthen translational applications.

## Figures and Tables

**Table 1:** An example of breakfast prepared for a participant. The calculated energy value of the breakfast for this participant was 845 kcal. GI values of the foods are indicated in parentheses [24,25].

Breakfast with High Glycemic Index (GI Value)		Breakfast with Low Glycemic Index (GI Value)	
White bread (75)	125 g	Whole wheat bread (49)	130 g
Raisin (65)	40 g	Apple (36)	300 g
White cheese (30)	90 g	White cheese (30)	90 g
Milk, full-fat (39)	260 mL	Milk, full-fat (39)	240 mL
Tomato (30)	200 g	Tomato (30)	200 g
**Energy** = 845 kcal		**Energy** = 845 kcal	
58% carbohydrate		58% carbohydrate	
17% protein		17% protein	
25% fat		25% fat	

**Table 2:** Overview of demographic and physiological characteristics of the participants (n = 10).

Characteristics	$\bar{X}$ ± SD
Age (year)	19 ± 1.5
Height (cm)	180.0 ± 0.10
Weight (kg)	76.2 ± 10.4
BMI (kg/m^2^)	23.9 ± 2.9
Fat mass (kg)	10.1 ± 6.3
Muscle mass (kg)	37.8 ± 3.8
Lean tissue mass (kg)	66.1 ± 6.5
VO_2_max (mL/kg/min)	49.0 ± 6.7
Resting heart rate (bpm)	59.7 ± 10.2
Target heart rate (bpm)	137.6–166.7 ± 17.3–15.5

BMI: body mass index, $\bar{X}$: mean, ±SD: standard deviation, VO_2_max: maximum oxygen consumption capacity.

**Table 3:** Blood glucose level measurements of participants and comparison of breakfast according to glycemic index value.

Blood Glucose Level (mg/dL)				
	**Breakfast with HGI**	**Breakfast with LGI**	**Z**	***p***
	$\bar{X}$ **± SD**	$\bar{X}$ **± SD**		
Fasting	84.3 ± 9.6	86.5 ± 5.6	−0.26	0.798
Pre-exercise	96.4 ± 9.3	78.0 ± 13.6	−2.30	0.022 *
Post-exercise	101.6 ± 10.7	84.4 ± 6.6	−2.81	0.005 **
**Blood Glucose Level (mg/dL)**				
	**Pre-exercise**	**Post-exercise**	**Z**	***p***
	$\bar{X}$ **± SD**	$\bar{X}$ **± SD**		
Breakfast with HGI	96.4 ± 9.3	101.6 ± 10.7	−0.67	0.506
Breakfast with LGI	78.0 ± 13.6	84.4 ± 6.6	−1.39	0.166

* *p* < 0.05 ** *p* < 0.01 Wilcoxon signed-rank test.

**Table 4:** Comparison of participants’ “3-Back test” results according to the glycemic index value of breakfast and pre and post-exercise.

3-Back Test	Breakfast with HGI	Breakfast with HGI	Z	*p*
	$\bar{X}$ ± SD	$\bar{X}$ ± SD		
**Percentage of Correct Answer (%)**				
Pre-exercise	35.6 ± 18.2	37.0 ± 15.3	−0.26	0.798
Post-exercise	48.5 ± 18.0	42.3 ± 15.6	−1.31	0.191
**Percentage of Wrong Answers (%)**				
Pre-exercise	19.4 ± 9.6	18.4 ± 8.54	−0.77	0.441
Post-exercise	18.5 ± 11.5	18.0 ± 9.0	−0.42	0.677
**RT_correct (ms)**				
Pre-exercise	572.6 ± 109.2	572.5 ± 183.7	−0.66	0.508
Post-exercise	515.0 ± 144.3	525.7 ± 159.5	−0.61	0.541
**RT_correct (ms)**				
Pre-exercise	549.6 ± 129.7	528.9 ± 162.9	−0.36	0.721
Post-exercise	516.1 ± 136.5	549.8 ± 120.7	−0.76	0.445
	**Pre-exercise**	**Post-exercise**	**Z**	***p***
	$\bar{X}$ **± SD**	$\bar{X}$ **± SD**		
**Percentage of Correct Answer (%)**				
Breakfast with HGI	35.6 ± 18.2	48.5 ± 18.0	−1.68	0.098
Breakfast with LGI	37.0 ± 15.3	42.3 ± 15.6	−0.97	0.331
**Percentage of Wrong Answers (%)**				
Breakfast with HGI	19.4 ± 9.6	18.5 ± 11.5	−0.97	0.333
Breakfast with LGI	18.4 ± 8.5	18.0 ± 9.0	−0.77	0.444
**RT_correct (ms)**				
Breakfast with HGI	572.6 ± 109.2	515.0 ± 144.3	−1.72	0.086
Breakfast with LGI	572.5 ± 183.7	525.7 ± 159.5	−1.17	0.241
**RT_correct (ms)**				
Breakfast with HGI	549.6 ± 129.7	516.1 ± 136.5	−1.00	0.314
Breakfast with LGI	528.9 ± 162.9	549.8 ± 120.7	−0.05	0.959

Wilcoxon signed-rank test, RT_correct: response time to correct answers; total: response time to all answers.

**Table 5:** Comparison of delta difference values in HbO_2_ increase in participants and breakfast according to glycemic index value and pre- and post-exercise.

HbO_2_-Delta_Difference (μmol/L)	Breakfast with HGI	Breakfast with LGI	Z	*p*
	$\bar{X}$ ± SD	$\bar{X}$ ± SD		
**Pre-exercise**	4.5 ± 2.3	3.0 ± 2.0	−1.84	0.066
**Post-exercise**	3.2 ± 2.3	3.9 ± 2.4	−0.178	0.859
**HbO_2_-delta_difference (μ** **mol/L)**	**Pre-exercise**	**Post-exercise**	**Z**	***p***
	$\bar{X}$ **± SD**	$\bar{X}$ **± SD**		
Breakfast with HGI	4.45 ± 2.32	3.20 ± 2.27	−1.68	0.093
Breakfast with LGI	2.98 ± 1.96	3.94 ± 2.41	−1.36	0.173

Wilcoxon signed-rank test, HbO_2_-delta_difference: delta difference in oxy-hemoglobin increase.

**Table 6:** Changes in blood glucose (mg·dL^−1^) at each time point and incremental area under the curve (iAUC) following high- and low-glycemic-index breakfasts.

Time Point (min)	$\mathrm{Breakfast}\;\mathrm{with}\;\mathrm{HGI}\;(\bar{X}$ ± SD)	$\mathrm{Breakfast}\;\mathrm{with}\;\mathrm{LGI}\;(\bar{X}$ ± SD)	Between-Condition Difference
**Fasting (0)**	84.3 ± 9.6	86.5 ± 5.6	–
**Pre-exercise (90)**	96.4 ± 9.3	78.0 ± 13.6	↑ HGI (*p* < 0.05) *
**Post-exercise (120)**	101.6 ± 10.7	84.4 ± 6.6	↑ HGI (*p* < 0.05) *
**Incremental AUC (0–120 min)**	622.5 mg·min·dL^−1^	0 mg·min·dL^−1^	↑ HGI (*p* < 0.01) **

* Significant difference between conditions (paired *t*-test). ** Incremental area under the curve (iAUC) computed by trapezoidal method using mean group values. ↑ means: HGI condition blood sugar level higher than LGI condition blood sugar level.

## Data Availability

The data presented in this study are available on request from the corresponding author. The data is not publicly available due to privacy reasons.
